# Systematic Differences in Signal Emitting and Receiving Revealed by PageRank Analysis of a Human Protein Interactome

**DOI:** 10.1371/journal.pone.0044872

**Published:** 2012-09-19

**Authors:** Donglei Du, Connie F. Lee, Xiu-Qing Li

**Affiliations:** 1 Quantiative Study Group, Faculty of Business Administration, University of New Brunswick, Fredericton, New Brunswick, Canada; 2 The Fu Foundation School of Engineering and Applied Science, Columbia University, New York, New York, United States of America; 3 Molecular Genetics Laboratory, Potato Research Centre, Agriculture and Agri-Food Canada, Fredericton, New Brunswick, Canada; Semmelweis University, Hungary

## Abstract

Most protein PageRank studies do not use signal flow direction information in protein interactions because this information was not readily available in large protein databases until recently. Therefore, four questions have yet to be answered: A) What is the general difference between signal emitting and receiving in a protein interactome? B) Which proteins are among the top ranked in directional ranking? C) Are high ranked proteins more evolutionarily conserved than low ranked ones? D) Do proteins with similar ranking tend to have similar subcellular locations? In this study, we address these questions using the forward, reverse, and non-directional PageRank approaches to rank an information-directional network of human proteins and study their evolutionary conservation. The forward ranking gives credit to information receivers, reverse ranking to information emitters, and non-directional ranking mainly to the number of interactions. The protein lists generated by the forward and non-directional rankings are highly correlated, but those by the reverse and non-directional rankings are not. The results suggest that the signal emitting/receiving system is characterized by key-emittings and relatively even receivings in the human protein interactome. Signaling pathway proteins are frequent in top ranked ones. Eight proteins are both informational top emitters and top receivers. Top ranked proteins, except a few species-related novel-function ones, are evolutionarily well conserved. Protein-subunit ranking position reflects subunit function. These results demonstrate the usefulness of different PageRank approaches in characterizing protein networks and provide insights to protein interaction in the cell.

## Introduction

The Google PageRank algorithm [1] provides high-quality search result rankings for websites [1] and journals [2]. In biology, the method can be used to rank proteins in protein networks [3]. Large-scale proteomics and yeast two-hybrid screenings [4] have recently generated large networks of proteins [5], [6], [7]. Because of the limitations of current proteomics technologies, most of these networks do not indicate signal/information-flow directions in protein interactions, or in some cases the information is only provided for a small sub-database such as the phosphorylation pathways, in spite of the fact that most protein-protein interactions in the cell are directional in terms of the information flow or the positions in pathways. Consequently, with the exception of some protein motif rankings [8], most PageRank and similar ranking approaches used for proteins also lack interaction directions [3], [9], [10], [11], [12].

Recently a large information-directional network of more than 2,000 human proteins as well as a database of more than 300 feedback-like pathway proteins were developed using protein interaction directional scores (PIDS) to predict the protein interaction direction of signal flow and pathways [13]. A positive PIDS for protein A to protein B in a signaling pathway means that the informational interaction direction is from A to B. The PIDS approach is innovative and useful for predicting the interaction direction between two proteins in pathways within the network. However, PIDS is not designed for describing each protein because very often a protein may be involved in more than one pathway or pathway branch and therefore can have various PIDS values. Therefore, protein ranking may provide useful information complementing the PIDS values. The PIDS values and PageRank values for this large, information-directional human protein database may be a useful reference for studying proteins in other species.

Several questions remain unanswered in protein ranking, including what the general difference in terms of the features of networking is between information emitting and receiving, whether high ranked proteins are more evolutionarily conserved than low ranked ones, and whether proteins with similar ranking tend to have similar subcellular locations. In this study, we use forward PageRank, reverse PageRank, and non-directional PageRank to rank proteins in the information-directional human protein network to answer those questions.

## Results

### Protein PageRank Order

The information/signal flow directions indicated by the positive PIDS values (>2.0) of the 2,249 human proteins and the 379 feedback-like pathway proteins [13] were used to construct a mathematical network. The proteins of this network were ranked using forward PageRank, reverse PageRank, and non-directional PageRank (See the Materials and Methods section). The ranking order of the 2,249 human proteins is listed in **File S1, File S2,** and **File S3,** for forward ranking, reverse ranking, and non-directional ranking, respectively. The ranking order of the 379 feed-back pathway proteins is listed in **File S4, File S5,** and **File S6,** for forward ranking, reverse ranking, and non-directional ranking, respectively. There is only a 2.9-fold difference in the average ranking value between the top 50 ranks and the lowest 50 ranks for the 379 feedback pathway proteins, while the difference is 13-fold for the 2,249 proteins.

### Distribution of Ranking Positions in Different Ranking Methods

**Figure 1:**
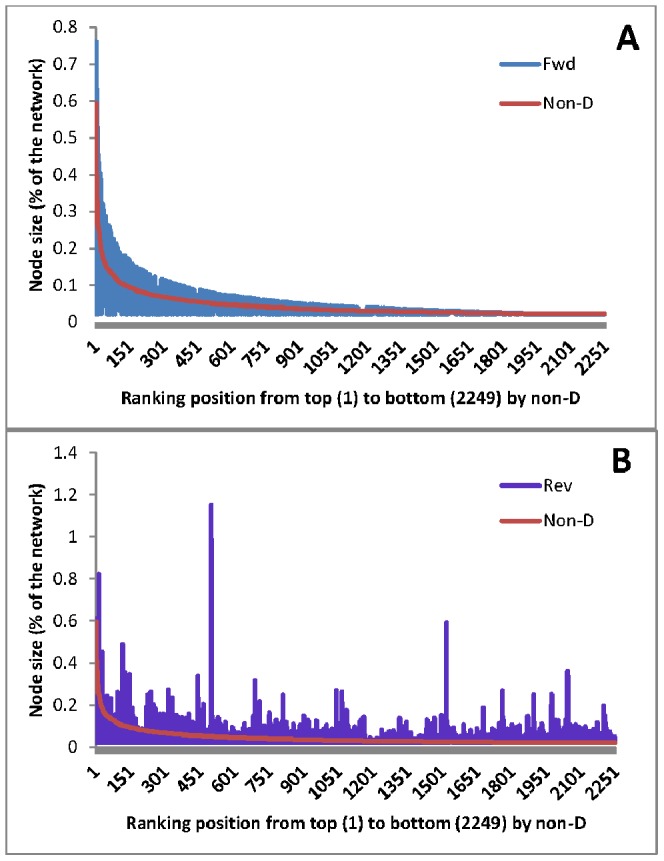
Ranking position distribution in forward method and reverse method when proteins are sorted by the non-directional ranking positions from top to bottom. A. Forward vs. non-directional. **B.** Reverse vs. non-directional. The node size is in terms of ranking percentage of the total ranking probability of all the proteins.

**Figure 2:**
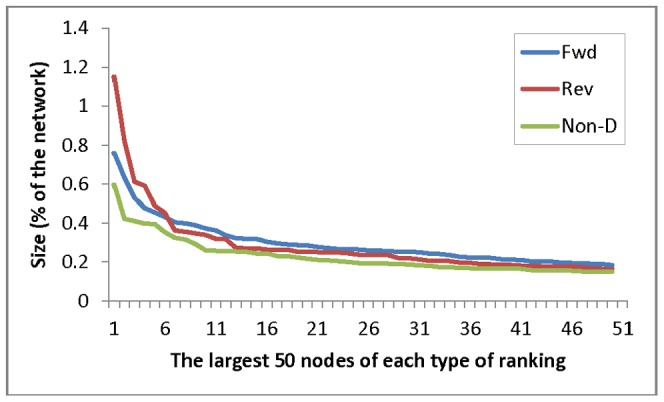
The relative sizes (reflected by % of ranking probability) of the 50 largest nodes (proteins) from each type of ranking of 2,249 human proteins in the interaction network. Fwd: Forward ranking, in which the larger a node is, usually the more sources it receives information from. **Rev:** Reverse ranking, in which the larger a node is, usually the more proteins it can regulate. **Non-D:** Non-directional ranking, in which the larger a node is, usually the more interactions (connections in the network) it has with others. Note that the first few large nodes of regulators (Rev) are much larger than those of the top information receivers (Fwd), but the remaining regular nodes are relatively smaller than receivers.

The ranking results of the 2,249 human proteins from forward ranking and non-directional ranking are highly correlated (r = 0.742, P = 0); whereas, the reverse ranking and non-directional ranking results have a much weaker correlation (r = 0.220, P<0.01). Forward and reverse rankings also have a weak degree of correlation (r = 0.269, P<0.01). When the proteins sorted by the non-directional ranking positions from top to bottom, the distribution of the forward ranking positions shows clear similarity to the non-directional ones (**Figure 1A**), but the reverse ranking has top ranked proteins scattered with a very different distribution to the non-directional ones although the ranking position tends to be high if the total interaction number of the protein is high (**Figure 1B**). The distribution of the top 50 ranked proteins from each ranking method showed that a few reverse ranking nodes are very large while the remaining proteins ranked lower than their counterparts in forward ranking (**Figure 2**).

### Top Information Emitters and Receivers

The algorithms in dealing with the information flow direction determine the following: the top ranked proteins in forward ranking are the top information receivers; the top ranked proteins in reverse ranking are the top information-emitters (i.e., regulators or senders); and the top ranked proteins in non-directional ranking are largely involved in the highest number of connections with other proteins.

The top 50, middle 50, and bottom 50 ranked proteins form the 2,249 human proteins are listed in **File S7, File S8,** and **File S9**, for forward, reverse, and non-directional rankings, respectively. The top ranked protein lists from the forward ranking and the non-directional ranking are relatively similar to each other, and very different from that of the reverse ranking (**File S10**). Among the 50 top ranked proteins from forward ranking, 29 proteins are identical to those from the top 50 in non-directional ranking but only 9 proteins are identical to the top 50 from the reverse ranked list (**File S10)**. Among the 50 top ranked ones, 8 are common among all three methods; each of them is involved in many pathways (**File S10; Table S1**). These eight proteins (ACVR1, CDC42, RAC1, RAF1, RHOA, TGFBR1, TRAF2, TRAF6) are evolutionarily very conserved (see e-value in the BLASTp search with *C. elegans* proteins (**File S10**)). These eight proteins are involved in many pathways, including signaling pathways, cell division, and cancer pathways (**File S10, Table S1**) and likely play very important roles in human cells. The results indicate that some broadest information-senders (reverse top) and some of the broadest information-receivers (forward top) can be the same proteins.

### Bottom-ranked Proteins

The bottom or low ranked ones from the 2,249 human proteins are nearly identical between the forward ranking and non-directional ranking, even though the order is different (**Files S7, S9**), and nearly completely different from the reverse ranking results (**File S8**). Therefore, in this human protein network, the bottom-ranked information-receivers are the proteins that have the fewest interactions with other proteins. However, the most specific regulators are not necessarily the proteins with the fewest interactions, which means that some proteins receive signals from different sources but regulate only one or very few specific proteins.

### Singling Pathways Involving the Top or Bottom Ranked Proteins

The majority (approximately 80–87%) of the 50 top ranked proteins from the 2,249 human proteins are signaling proteins in all three rankings (forward, reverse, and non-directional), while there are very few signaling pathway proteins for low ranked ones (**Table 1**). The most frequent signaling pathway for the top-ranked proteins is the mitogen-activated protein kinase (MAPK or MAP kinase) pathway, which accounts for approximately 24–34% of proteins among the top 50 proteins in all three rankings (**Table 1**). MAPK signaling pathway proteins tend to be more frequent in the top 50 proteins in forward ranking than those in non-directional ranking (**Table 1** **)** (P = 0.09, but not reached P<0.05 in ChiTest).

**Table 1:** Categories of top 50 ranked proteins.

Ranking method	No. ranked proteins	Proteins in known pathways	Proteins without pathway assignment	Signalling proteins (no.)*	Signalling/knownpathway proteins (%)	MAPK pathway proteins (no)*	MAPK/all proteins (%)
Forward	Top 50	43	7	37	86.0	15	34.9
Reverse	Top 50	46	4	39	85.0	14	30.4
Non-directional	Top 50	45	5	37	80.4	11	24.4
Forward	Bottom 49^a^	17	32	7	14.29	1	5.88
Reverse	Bottom 50	21	29	11	22.00	2	9.52
Non-directional	Bottom 49^b^	17	32	7	14.29	1	5.88

* : The ChiTEST analysis comparing forward top ranks and reverse top ranks with non-directional top ranks showed no significant difference (P<0.05) in either the number of signalling proteins or the number of MAPK proteins.

a one of the bottom 50 proteins did not found BLASTp target in *C. elegans*.

b because one of the bottom 50 proteins could not be detected in KEGG database.

Although MAPK pathway proteins are most frequent in top ranked ones, interestingly the highest ranked protein is CBL, which is an ErbB signaling pathway protein and a growth factor receptor (**File S8**). The top position in reverse ranking suggests that this protein as well as its represented pathway plays a key regulation role in the human protein interactome.

Among the top ranked non-signaling, non-pathway proteins, actin (involved in skeletal muscle) is one of the most common proteins; it is represented by two top ranked proteins in the forward ranking, zero in the reverse ranking, and five top ranked proteins in the non-directional ranking (data not shown). Ubiquitin B (involved in non-lysosomal intracellular protein degradation) and rhophilin (Rho GTPase binding protein) are also among the top ranked proteins in reverse ranking.

The majority of the low ranked proteins do not belong to any known pathways, and have very few MAPK pathway proteins. Low ranked proteins are protein categories that are functionally different from top-ranked ones. For example, cyclin, a group of proteins specifically receiving signals for the initiation of cell division, appears frequently among the 50 low ranked proteins in the reverse rankings (the CCN proteins at the bottom in **File S2**). The coincidence of its presence in bottom ranked positions in reverse ranking and absence in the bottom rankings in forward ranking suggests that cyclins receive relatively broad sources of stimulus/signals for cell division but direct relatively few targets to allow DNA duplication and cell division.

### Evolutionary Conservation

The top 50 ranked proteins from each of the forward, reverse, and non-directional rankings of the 2,249 human proteins were compared by running a BLASTp search against *C. elegans* proteins in the KEGG database (www.genome.jp/kegg/). The average number of BLASTp bits of the top 50 proteins was significantly different between the ranking methods. However, the average length, in number of amino acids, of the 50 top ranked proteins in the non-directional ranking was shorter than that in the forward or reverse rankings (**Table 2**).

**Table 2:** Comparison of the ranking methods in terms of BLASTp results and the average length of the 50 top ranked proteins.

Rankingmethod	No. of toprankedproteins	BLAST bitsaverage^a^	Protein length average(No. of amino acids)
Forward	50	322.58 A	686 A
Reverse	50	329.58 A	699 A
Non-directional	50	280.54 A	506 B

a values labelled with the same letter in the same column are not significantly different at the P<0.05 level based on ANOVA and Duncan’s multiple-range test.

The most frequently connected proteins in the non-directional ranking tend to be smaller (on average, 506 amino acids, i.e. a.a) than those in the forward (686 a.a.) and reverse (699 a.a.) rankings (**Table 2**). There was no significant difference between the ranking methods in terms of the BLAST bits (**Table 2**).

For the 2,249 human proteins, the top 50, middle 50, and bottom 50 proteins were compared to their counterparts in the nematode *C. elegans* using a BLASTp search. In forward and reverse rankings, the top ranked proteins were more conserved in terms of BLASTp bits than the middle or low ranked proteins in both species (**Table 3**). This difference between the ranking methods is not attributable to a difference in protein size (amino acid number), since protein length does not differ significantly between the top, middle, and low ranked protein groups in forward ranking or reverse ranking (**Table 3**).

**Table 3:** Ranking position, degree of evolutionary conservation in terms of BLASTp hit bits in a search against *Caenorhabditis elegans* proteins, and protein length.

Ranking method	Ranking degree	No. of top rankedproteins	BLAST bits average^a^	Average protein length(No. of amino acids)
Forward	Top	50	322.58 A	686 A
	Mid	49	200.16 B	712 A
	Low	49	197.90 B	734 A
Reverse	Top	50	329.58 A	699 A
	Mid	50	200.04 B	628 A
	Low	49	156.80 B	604 A
Non-directional	Top	50	280.54 A	506 B
	Mid	50	292.70 A	872 A
	Low	50	203.06 A	734 AB

a values labelled with the same letter in the same column, within each top, middle or bottom panel, are not significantly different at the P<0.05 level based on ANOVA and Duncan’s multiple-range test.

The non-directional ranking showed a similar tendency with respect to BLASTp bits as the forward and reverse ranking although it did not reach a significant level (bits value: 292.70, 280.54, and 203.06 for top, middle and low ranks, respectively) (**Table 3**). Protein length (505.5 a.a.) in non-directional top rankings was shorter than that (871.8 a.a.) in the non-directional middle rankings (**Table 3**).

A few top ranked proteins had very low evolutionary conservation between human and *C. elegans*, and interestingly these a few proteins all belong to novel functions specific to humans, compared to *C. elegans*. In the forward ranking, those human proteins without a closely related *C. elegans* counterpart are T cell receptor signaling pathway proteins (has:919), Fc fragment of IgE (has:2207), and a v-rel protein (hsa:5970) (**File S7**). These proteins likely evolved after the separation between the human ancestors and *C. elegans* ones.

### Ranking Effectiveness Related to Subcellular Locations of Proteins

For the 379 feedback pathway proteins that have subcellular location information, the ranking results for subcellular location information are listed in **File S4**, **File S5**, and **File S6**, for the forward, reverse, and non-directional rankings, respectively. The 50-top ranked proteins in forward ranking and the 50 lowest ranked ones in reverse ranking were all non-nucleus ones (**Table 4**). In non-directional PageRank ranking, the 50 top ranked proteins comprised 3 nucleus ones and 47 others (**Table 4**). Overall, all three types of ranking (non-directional, forward, and reverse) showed clearly improved grouping of the proteins than the PIDS approach in terms of subcellular location. The directional rankings (forward and reverse) were more effective in grouping proteins based on subcellular locations than the non-directional ranking (**Table 4**).

**Table 4:** Comparison of protein location grouping by the PIDS approach and the protein ranking approach using feedback pathway proteins*.

Sorting criterion	No. proteins	Nucleus proteins (n)
Highest PIDS	50	25
Lowest PIDS	50	20
Top rank (forward)	50	0
Low rank (forward)	50	31
Top rank (reverse)	50	40
Low rank (reverse)	50	0
Top rank (non-directional)	50	3
Low rank (non-directional)	50	31

*The protein database analyzed is the database of 379 feedback pathway proteins in the previous publication [13]. The PIDS values and subcellular location information were counted according to the same publication [13]. The ranking data are from the present study.

For the 2,249 human proteins, the subcellular locations of proteins are not as clearly separated by different ranking methods as for the feedback pathway proteins alone, likely because this 2,249 protein database is non-selective and includes both feedback and non-feedback pathway proteins. Nevertheless, for this mixed 2,249-protein database, the protein ranking position patterns of the top 50 and bottom 50 proteins are still clearly different between the forward ranking and the reverse ranking (**Table 5**). This difference between the two directional-ranking methods is likely due to the predominance of non-feedback pathway proteins in this 2,249 protein database. Nucleus proteins were found to be more frequent in top 50 forwardly-ranked proteins than bottom 50 forwardly-ranked proteins (38 vs. 18 proteins, respectively) (**Table 5**). This pattern is opposite to that of the reversely-ranked proteins. Of the cellular membrane proteins, the distribution between top and bottom ranked positions is opposite to that of the nucleus proteins (**Table 5**).

**Table 5:** Subcellular locations of top- and bottom- ranked proteins from the 2,249 protein database that have both feedback and non-feedback pathway proteins^a^.

	Subcellular location^b^			
Ranked position	Nucleus (%)	Cytoplasm (%)	Membrane (%)	In both nucleus and another (%)
Forwardly ranked top 50	38	58	46	36
Forwardly ranked bottom 50	18	28	58	12
Reversely ranked top 50	48	78	50	42
Reversely ranked bottom 50	52	56	32	28
ChiTEST^c^ (P)	0.0026**	0.1068NS	0.0309*	0.0404*

a The 2,249 protein database [13] analyzed contains both feedback and non-feedback pathway proteins.

b Protein location based on http://www.uniprot.org/. Each protein can be in more than one location. Each ranked position group (top 50 or bottom 50) contains 50 proteins that their subcellular locations can be identified or suggested by the UniProt database. In case 1 or 2 proteins lack the subcellular location information, the proteins at the 51^st^ and 52ed positions were used as replacements.

c The ChiTEST was between forward ranking and reverse ranking. The top/bottom ratio of protein numbers of the reverse ranking was tested using the top/bottom ratio of forwardly ranked proteins as the reference ratio.

* Significant.

** Highly significant. NS: Non-significant.

Interestingly, regardless of forward or reverse ranking, the top ranked proteins have more cytoplasmic proteins and more proteins with multiple locations (i.e, in nucleus and other locations) than the bottom ranked ones. The dominance of cytoplasmic proteins in top ranked ones is likely because the cytoplasm is a dual location that is internal compared to the membranes, but is external compared to the nucleus. The high occurrence of multiple location proteins in top ranked ones (**Table 5**) is consistent with the finding that some proteins are among the top ranked ones in both forward and reverse rankings (**Table S1**). These results suggest that feedback pathway proteins and non-feedback pathway proteins have certain general differences for the relationship between ranked positions and subcellular locations. For this mixed database, which consists mainly non-feedback pathway proteins, the top forwardly-ranked proteins are mainly internal proteins that receive signals to do work, and the top reversely-ranked proteins are mainly external proteins (e.g. membrane or cytoplasmic proteins vs nucleus ones) that emit signals.

The analyses of both the 379 feedback pathway protein database and the 2,249 total protein database demonstrated that proteins with similar ranking positions tend to have similar subcellular locations.

### Member-specific Position in Ranking for Family Proteins

It is known that members of family proteins do not always have identical domains and functions [14]. Therefore, tools are needed to distinguish protein members. In this study, protein ranking appears to be effective in distinguishing members of the same protein family.

Tyrosine phosphatases are a group of enzymes that remove phosphate groups from phosphorylated tyrosine residues on proteins, which is a common post-translational modification affecting protein stability and regulating enzyme activity. These enzymes are important regulators in cell division, cell growth, proliferation, differentiation and transformation [15], [16]. In the reverse ranking in this study, two tyrosine phosphatases ranked in the top 50 proteins, 4 in the middle 50 proteins and two in the bottom 50 proteins according to their regulatory role played in the whole network. (**Table S8**). This function-specificity-based sorting provides information that otherwise cannot be obtained by simply phylogenetic studies of this gene/protein family.

Rab proteins, which are peripheral membrane proteins and belong to a superfamily of small GTPase, function as address labels for defining the protein identity in vesicle trafficking [17]. It is expected that the specificity of each GTPase has to be under tight control. However, only about 60–70 Rab proteins have been identified in the human genome. An unanswered question is how these 60–70 Rab proteins distinguish and assist the vesicle trafficking of so many human proteins, while ensuring specificity. In the forward ranking, 8 Rab proteins (RAB7A, RAB6B, RAB38, RAB34, RAB33B, RAB21, RAB13, and NKIRAS1) are among the bottom ones, confirming that they are under very specific control (**File S7**). These 8 Rab proteins belong to the same large branch on the Rab protein phylogenetic tree [17]. However, it is interesting that 5 potential Rab proteins (according to BLASTp results) ranked in the top 50 in the same forward ranking, suggesting that these 5 Rab proteins receive regulation from many sources (**File S7**). These 5 proteins are CDC42, HRAS, RAC1, and TARF2 and all have high similarity to *C. elegans* Rab proteins in BLASTp search (**File S7**). According to these results, some Bab proteins assist the vesicle trafficking of very few proteins while others can assist many. It would be interesting to investigate the common features of these “many proteins” that can be assisted by the top ranked Rab proteins.

However, theoretically, not all the proteins that are ranked on the bottom are guaranteed to be narrowest regulators or information emitters, because it might be simply due to the lack of thorough studies of those particular proteins. Future ranking of larger human protein databases and other mammal protein databases when they are available can adjust the ranking positions of these proteins.

## Discussion

### General Difference in Information Emitting and Receiving in Human Cells

In this study, the ranking results between forward ranking and non-directional rankings are more similar than those between reverse ranking and non-directional ranking. Since the non-directional ranking is largely a reflection of the number of interactions, the strong correlation between the forward and non-directional ranking would suggest that the increased degree of information receiving is achieved largely through the increased number of proteins interactions. It is likely that most information receivers are the proteins who are the workers that convey the specific tasks in the cell. The lack of a strong correlation in ranking results between reverse ranking and non-directional ranking and the very high ranking of very few proteins might suggest that the key regulators do not necessarily issue information directly to many proteins. Similar to the president who issue orders usually to key executives only, key regulators in human protein networks do not have to directly communicate with many proteins. However, this does not mean that the top ranked proteins in reverse ranking are very specific in interaction. For example, the top ranked protein PLCG1 in forward ranking (**File S1**) is known to be involved in 19 KEGG pathways (http://www.genome.jp/dbget-bin/www_bget?hsa:5335) while the top ranked protein CBL in reverse ranking (**File S2**) is also involved in 9 pathways, including the regulation of the vascular malformation/cancer pathway of PI3K (PIK3CA) –PTEN – PKB/AKT – mTOR (http://www.genome.jp/dbget-bin/www_bget?hsa:867). The second top ranked protein TRAF6 (hsa:7189) in reverse ranking is involved in 20 pathways (http://www.genome.jp/dbget-bin/www_bget?hsa:7189 ). The general difference in **Figure 1A** (forward ranking) and **Figure 1B** (reverse ranking) only means that the ranking position in reverse ranking does not rely as heavily as the forward ranking does on the total number of interactions. If similar results can be confirmed in other signal-directional databases of proteins, this general difference in information emitters and receivers might provide insights into signal-related biology.

### Categories of Top Ranked and Low Ranked Proteins

The protein database [13] used in this study has predicted information directions of their protein-protein interactions. The directions are based on PIDS values which were determined using known information of the proteins such as conserved domains and pathway directions [13]. PIDS value prediction was strongly supported by the known signaling pathway literature with an 87.5% accuracy [13]. Protein-protein interaction in signal transduction pathways such as phosphorylation cascades is clearly directional in terms of information/signal flow. The information flow between proteins is not so straightforward for some other proteins such as actins and other structural assemblies of protein complexes. However, the time order or step by step influence in other types of pathways might also be a type of informational direction in a broad sense. This is because the ranking position of a protein is an overall consideration of the entire network, not only based on the direct interactions linked to that given protein. This overall evaluation in ranking can explain why actins and IgE fragments are ranked high in forward ranking. The database [13] with PIDS values in protein-protein interactions is a signaling protein-enriched database, which might partly explain why signaling pathway proteins are most frequent in the top ranked proteins. However this cannot explain why signaling pathway proteins are not frequent in bottom ranked proteins and why some non-signaling pathway proteins such as actin and IgE proteins also ranked high. Likely, the knowledge learned from this study might not only have insights into the function of signaling networks but also to a certain degree into the general protein-protein interaction network in the human cell.

In this study, the top ranked proteins are mainly members of signaling pathways, particularly the MAPK pathway. MAP kinases (MAPK) are serine/threonine-specific protein kinases that respond to extracellular stimuli [18], [19] and regulate various cellular activities, including gene expression, cell division, differentiation, proliferation, and cell survival/apoptosis [20]. The ranking results fit the MAPK functions because the top forward rankings indicate that MAP kinases are very active receivers of signals (stimuli), and the top reverse ranking indicates that some MAP kinases are very active regulators or signal senders to other proteins. Several MAP kinases are top ranked in both forward and reverse ranking, suggesting that these proteins themselves are both active signal receivers and active regulators.

### Ranking, Evolutionary Conservation, “Old Genes” and “New Genes”

Not all proteins contribute equally to organismal fitness [21], and an understanding of both sequence evolution and functional/phenotypic evolution is necessary. In this study, the BLASTp analysis results suggest that top ranked proteins tend to be more conserved than other proteins in terms of BLASTp bits compared with proteins of the nematode *C. elegans*, an evolutionarily distant species (**Table 3**). It is not surprising that top ranked proteins are evolutionarily more conserved because mutations of such proteins can interfere with many other cellular activities. For example, the MAPK pathway acts as an "on" or "off" switch by communicating signals from receptors on the cellular surface to the DNA in the nucleus, and MAP kinase mutation often causes the development of cancers [20], [22]. Since eight proteins are common to the 50 top-ranked proteins from all three ranking approaches, it is clear that new species tend to use evolutionarily conserved proteins for multiple functions.

However, among the proteins ranked on top in forward ranking, a few proteins belong to actins, T-cell receptors, and IgE fragment proteins that are specific to humans and higher animals but not needed or do not exist in *C. elegans* (**File S4**). Muscle actins, T-cell receptors, and antibody proteins are obviously important to human health. The relatively high ranking of these new proteins in forward ranking suggests that they are heavy-tasked “workers” responding to multiple signals directly or indirectly. It would be interesting to investigate the evolutionary pathway that led humans and other higher animals to assign multiple such important functions to new proteins/genes. An understanding of the functional mechanisms underlying this ranking difference might allow us to better understand the evolution of higher animal species.

For the 379 feedback-like pathway proteins [13], their directional ranking sorted the subcellular locations well but did not show significantly greater evolutionary conservation compared to the low ranked proteins in ether forward or reverse ranking (data not shown). This may be due to the fact that the feed-back population size was too small or that the upstream and downstream proteins in the feedback pathways are more or less equally important and they closely cooperate to perform a function. Further investigation is needed to verify whether feedback pathways are relatively short and lack large nodes in a protein network.

### Ranking Positions and Subcellular Locations of Proteins

For the feedback pathways, 40 of the top 50 reversely ranked ones are nucleus ones, but no nucleus proteins in the bottom ranked ones (**Table 4**). For the 2,249 total protein database which is dominated by non-feedback proteins, the subcellular distribution pattern (**Table 5**) of top- and bottom- ranked proteins is approximately opposite to that of the feedback pathway proteins, a result as expected. The ranking of the feedback proteins (**Table 4**) and the total protein database proteins (**Table 5**) in relation to subcellular location clearly indicates that PageRank ranking of proteins can effectively group proteins into similar subcellular locations. Interestingly, the location information was not used in the ranking calculation in the present study. We found a relationship between ranking position and both subcellular location and degree of evolutionary conservation. From a statistical standpoint, further research is required to determine whether proteins that cooperate to perform a certain function tend to be present in the same subcellular location and/or have a similar degree of evolutionary conservation.

Signals generated in response to extracellular stimuli at the plasma membrane are transmitted through cytoplasmic transduction cascades to the nucleus. An example is the endocytosis Rab5-APPL signal transduction pathway. Rab5, a small GTPase, is localized to the plasma membrane and early endosome and functions as a key regulator of vesicle trafficking during early endocytosis [23]. Rab5 regulates multiple proteins [24]. The APPL nucleocytoplasmic shuttling is controlled by Rab5 [24]. Both APPL1 and APPL2 are essential for cell proliferation and their function requires Rab5 binding [25]. APPL translocates from the membranes to the nucleus where it interacts specifically with the nucleosome remodeling and histone deacetylase multiprotein complex [25]. In the present study, this Rab5 (RAB5A), which is upstream, a relatively broader regulator than APPL, and located in cytoplasm, was ranked relatively high (P = 0.003332). Whereas, both APPL1 and APPL2, which are downstream in the Rab5-APPL pathway and are located in nucleus, were ranked relatively low (P = 0.001799) in the forward ranking of feedback pathway proteins (**File S4**).

### Utilization of the Ranking Position Information

The information acquired in this study may be helpful for future investigations in various areas. The interpretation of the ranking results suggests that the protein PageRank rankings, particularly the directional rankings, can be informative approaches for the characterization of protein networks. Similar and improved ranking approaches can be used to rank the proteins of other species when their information-directional interactomes are available. MAPK pathways contain many proteins and need to be analyzed using systematic/network approaches such as this overall ranking. The ranking position of pathway members in different types of ranking (forward, reverse, and non-directional) in this study provide insights into which members are likely to be mainly signal receivers, signal senders (regulators), or both regulators and regulated members. Similarly the directional ranking approaches can be used in predicting the functional difference between subunits of the same protein complex and therefore assist the identification of targets during pharmaceutical drug development.

Some broadest information-senders and some of the broadest information-receivers are the same proteins, and therefore can be ranked in top positions in both forward and reverse rankings. This information can be very helpful for narrowing down the short list of proteins for various genetic studies, including genetic manipulations, and old/new genes that have essential functions. Since the human protein database can serve as a reference for potential functions of proteins in other species, the top ranked proteins and the findings from this study can be candidates for many specific biological studies in humans and other species.

Another practical use of protein directional ranking information is to predict the likely subcellular location and pathway location (upstream or downstream) of proteins, or combined with other protein location prediction approaches to improve the prediction likelihood. For feedback pathway proteins, it is known from **Table 4** that none of the bottom ranked proteins in reverse ranking is a nucleus protein. For other proteins, we may expect that they are distributed differently from the feedback pathway proteins. We did literature search of the bottom 10 proteins in reverse ranking of the 2,249 proteins (**File S8**) and confirmed, as expected, their nucleus localization for the proteins TNS4 [26], SUPT6H [27], ALX and HSH2D [28], ARF3 [29], and Nek9 (at least a small portion located in the nucleus) [30]. Learned from **Table 5**, it is also known that top ranked proteins, regardless of in forward ranking or reverse ranking, have more possibility to be located in the cytosol or in both nucleus and a non-nucleus place than the bottom ranked ones.

### Conclusion and Outlook

Overall, the reverse ranking results are not a simple backward reading of the forward ranking results in directional protein PageRank. The non-directional ranking value of a protein is not the mean value of forward and reverse rankings. The signal emitting/receiving system is characterized by key-emittings and relatively even receivings. Various top information-emitters are also top information-receivers. Key regulators do not necessarily interact directly with a huge number of proteins. Signaling pathway proteins are the most frequent type of proteins in top-ranked ones out of the three ranking methods (forward, reverse and non-directional). Directional PageRank ranking of proteins can be more effective than non-directional ranking in sorting and estimating proteins in terms of subcellular location. The ranking positions may help select proteins for experimental studies. Top-ranked proteins tend to be conserved to a greater extent during evolution than the low ranked ones in directional ranking. Since the mathematical ranking calculation was completely independent of the evolutionary conservation and subcellular analysis, the concordance found between them suggests a biological relationship between ranking position and evolutionary conservation as well as subcellular location.

## Materials and Methods

### Protein Database

The directed interactions of the 2,249 human proteins and 379 feedback-like pathway proteins are based on a previous study by Liu et al. [13]. The signal flow direction is indicated by a positive PIDS value (>2.0) [13], which is essentially a probability calculated based on several factors [13]. We used the signal flow direction estimated by PIDS value qualitatively (i.e., forward or reverse) and treated all interactions equally in constructing the adjacency matrix, a mathematical network. The nodes of the network represent the proteins. There is a directional link from protein A to protein B whenever the pathway protein A to protein B had a positive PIDS value (>2.0) in the Liu et al. database. We then applied a PageRank analysis of this network to obtain the ranking of the proteins based on the interaction among the proteins.

### Protein PageRank Method

The PageRank approach is explained as follows. Let A be the adjacency matrix of the protein network. Let N be obtained from A by dividing each entry in A by its out-degree. Then the basic PageRank (PR) satisfies.
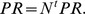
Where PR is the eigenvector of matrix *N^t^* (the transpose of A), corresponding to the eigenvalue 1.

In the scaled version of PageRank, let 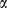
be a scale factor (a.k.a. damping factor) between 0 and 1. We use a new matrix 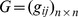
to replace 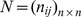 as follows:
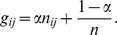
Where n is the dimension of A.

Hence, the scaled PageRank (PR) satisfies.
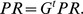



The reverse PageRank can be obtained analogously by using the transposed adjacency matrix
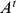
instead of A in the above calculation.

The ranking of proteins was based on the forward direction according to PageRank (forward ranking) [1], reverse direction according to PageRank (reverse ranking) or average ranking (non-directional ranking), with a damping factor (a residual probability, scale factor alpha) of 0.85 in accordance with the default setting in PageRank [1].

In the undirected PageRank, the adjacency matrix is symmetric and hence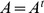, leading to the same forward and backward PageRank. The only change in the above calculation is N, where the out-degree is replaced with the in-degree. The Matlab code for the protein ranking is available for download from http://people.unb.ca/~ddu.

### Difference in Forward and Reverse Rankings

In forward ranking, top ranked proteins (also known as “authorities” or prestige in social network analysis) are always big nodes in the network even though this type of ranking identifies the protein nodes that receive signals. In reverse ranking, top ranked proteins (also known as “hubs” in social network analysis) are those that emit signals. Low ranked proteins are ones that do not receive much interaction from other proteins in forward ranking, and proteins that do not initiate interaction in reverse ranking.

The essential difference between forward and reverse rankings can be explained easily by making an analogy based on the PageRank algorithm. Imagine an explorer who is randomly browsing the protein network. In forward ranking, the explorer, starting at a random protein, picks each outgoing link with equal probability and follows the links for a sequence of a certain number of steps, say *k* steps. At each step, he picks a random outgoing link from his current position and follows it to where it leads. If there are no outgoing links, he just stays where he is. Based on Markov theory, this random walk within the protein network is a Markov chain, and except in certain degenerate special cases, it can be proved that the PageRank values of all nodes converge to limiting values as the number of update steps *k* goes to infinity. This gives a final PageRank value for each node, which is equal to the reciprocal of the expected number of steps taken to reach that node, starting from a randomly chosen one. Therefore, a node with a smaller expected number of steps to be taken by the explorer is given a higher rank. In the implementation of PageRank, an extra scaling factor is utilized to deal with the degenerate cases and ensure the uniqueness of the PageRank value.

The signal flow direction can be determined qualitatively as forward or reverse by the probability-like PIDS values [13]. It is unclear scientifically at this stage whether and how this PIDS information can also be used quantitatively (i.e., adjust each interaction with an estimation about whether the signal flow direction is very likely or less likely). If the signal flow direction is used quantitatively, the optimal damping factor in PageRank of this protein database should be the one that can give the most robust and meaningful ranking results. However, this would require extensive numerical test computation for different choices of alpha and data interpretation. This is why we used the default setting of damping factor (0.85) in PageRank [1] in this first study and leave the extensive numerical tests for future studies.

In the reverse PageRank ranking, we simply replace the outgoing link at each step with an incoming link. Given that for any given node, these two sets of links are not identical, the final PageRank values are different.

Ranking position is based on the global evaluation of the network, not purely determined by the direct interactions. In forward ranking, higher values are givens to proteins that receive signals from large nodes. Similarly, in reverse ranking, higher values are given to proteins that send information to large nodes. Therefore, a protein ranked high is not necessarily a protein having many connections in the network.

### BLASTp Search Against a Protein Database of a Genetically Distant Species

A search related to the top ranked, mid-ranked, and low ranked proteins (50 proteins each) was then performed using the Basic Local Alignment Search Tool (BLASTp search) [31] against the NCBI nematode (*Caenorhabditis elegans*) protein database on the website http://www.genome.jp/kegg/kegg2.html. *C. elegans* was selected for this comparison because its protein database is one of the most complete in existence. ChiTEST, ANOVA and Duncan’s multiple-range test were conducted to compare the ranking results using both Excel 2010 and SAS 4.3.

## Supporting Information

Table S1 **The eights proteins ranked on top 50 by all three methods–forward, reverse, and non-directional.**
(DOCX)

File S1 **Total proteins_Forward ranking results.xls.**
(XLS)

File S2 **Total proteins_ Reverse ranking results.xls.**
(XLS)

File S3 **Total proteins_ Non-directional ranking results.xls.**
(XLS)

File S4 **Feedback proteins_Forward ranking results.xls.**
(XLS)

File S5 **Feedback proteins_Reverse ranking results.xls.**
(XLS)

File S6 **Feedback proteins_Non-directional ranking results.xls.**
(XLS)

File S7 **Total proteins_Forward ranking_Top, mid, and bottom 50.xls.**
(XLS)

File S8 **Total proteins_Reverse ranking_Top, mid, and bottom 50.xls.**
(XLS)

File S9 **Total proteins_Non-D ranking_Top, mid, and bottom 50.xls.**
(XLS)

File S10 **Total proteins_Fwd and Rev Shared top ranked proteins.xls.**
(XLS)
