# Supplementary material for: Systematic Differences in Signal Emitting and Receiving Revealed by PageRank Analysis of a Human Protein Interactome
Source: PLoS One. 2012 Sep 19;7(9):e44872. doi: 10.1371/journal.pone.0044872 (PMC3446998; doi:10.1371/journal.pone.0044872)
Supplement: Table S1 — The eights proteins ranked on top 50 by all three methods–forward, reverse, and non-directional. (DOCX) [file pone.0044872.s001.docx]

**Table S1.** The eights proteins ranked on top 50 by all three methods—forward, reverse, and non-directional.

| Protein Name*^a^* | Kegg ID*^b^* | IPI Number | Gene name | Definition | Pathway | Bits | E-value | a.a. No. |
| --- | --- | --- | --- | --- | --- | --- | --- | --- |
| ACVR1 | hsa:90 | IPI00029219 | ACVR1, ACTRI, ACVR1A, ACVRLK2, ALK2, FOP, SKR1, TSRI | activin A receptor, type I | Cytokine-cytokine receptor interaction; TGF-beta signaling pathway. | 242 | 2.00E-64 | 509 |
| CDC42 | hsa:998 | IPI00007189 | CDC42, CDC42Hs, G25K | cell division cycle 42 (GTP binding protein, 25kDa) | MAPK signaling pathway;  Chemokine signaling pathway; Endocytosis;  Axon guidance;  VEGF signaling pathway;  Focal adhesion;  Adherens junction; Tight junction; T cell receptor signaling pathway; Fc gamma R-mediated phagocytosis; Leukocyte transendothelial migration; Neurotrophin signaling pathway; Regulation of actin cytoskeleton; GnRH signaling pathway; Bacterial invasion of epithelial cells; Epithelial cell signaling in Helicobacter pylori infection; Pathogenic *Escherichia coli* infection; Shigellosis; Salmonella infection; Pathways in cancer; Renal cell carcinoma; Pancreatic cancer. | 353 | 2.00E-98 | 191 |
| RAC1 | hsa:5879 | IPI00010271 | RAC1, Rac-1, TC-25, p21-Rac1 | ras-related C3 botulinum toxin substrate 1 (rho family, small GTP binding protein Rac1) | MAPK signaling pathway; Chemokine signaling pathway; Phagosome; Wnt signaling pathway; Axon guidance; VEGF signaling pathway; Osteoclast differentiation; Focal adhesion; Adherens junction; Toll-like receptor signaling pathway; Natural killer cell mediated cytotoxicity; B cell receptor signaling pathway; Fc epsilon RI signaling pathway; Fc gamma R-mediated phagocytosis; Leukocyte transendothelial migration; Neurotrophin signaling pathway; Regulation of actin cytoskeleton; Pancreatic secretion; Amyotrophic lateral sclerosis (ALS); Bacterial invasion of epithelial cells; Epithelial cell signaling in Helicobacter pylori infection; Shigellosis; Salmonella infection; Pathways in cancer; Colorectal cancer; Renal cell carcinoma; Pancreatic cancer; Viral myocarditis. | 306 | 4.00E-84 | 192 |
| RAF1 | hsa:5894 | IPI00021786 | RAF1, CRAF, NS5, Raf-1, c-Raf | v-raf-1 murine leukemia viral oncogene homolog 1 | MAPK signaling pathway; ErbB signaling pathway; Chemokine signaling pathway; Vascular smooth muscle contraction; VEGF signaling pathway; Focal adhesion; Gap junction; Natural killer cell mediated cytotoxicity; T cell receptor signaling pathway; B cell receptor signaling pathway; Fc epsilon RI signaling pathway; Fc gamma R-mediated phagocytosis; Long-term potentiation; Neurotrophin signaling pathway; Serotonergic synapse; Long-term depression; Regulation of actin cytoskeleton; Insulin signaling pathway; GnRH signaling pathway; Progesterone-mediated oocyte maturation; Melanogenesis; Tuberculosis; Hepatitis C; Influenza A; Pathways in cancer; Colorectal cancer; Renal cell carcinoma; Pancreatic cancer; Endometrial cancer; Glioma; Prostate cancer; Melanoma; Bladder cancer; Chronic myeloid leukemia; Acute myeloid leukemia; Non-small cell lung cancer. | 360 | 1.00E-99 | 648 |
| RHOA | hsa:387 | IPI00478231 | RHOA, ARH12, ARHA, RHO12, RHOH12 | ras homolog gene family, member A | Chemokine signaling pathway; Endocytosis; Vascular smooth muscle contraction; Wnt signaling pathway; TGF-beta signaling pathway; Axon guidance; Focal adhesion; Adherens junction; Tight junction; T cell receptor signaling pathway; Leukocyte transendothelial migration; Neurotrophin signaling pathway; Regulation of actin cytoskeleton; Pancreatic secretion; Bacterial invasion of epithelial cells; Pathogenic Escherichia coli infection; Pertussis;  Tuberculosis; Pathways in cancer; Colorectal cancer. | 344 | 1.00E-95 | 193 |
| TGFBR1 | hsa:7046 | IPI00005733 | TGFBR1, AAT5, ACVRLK4, ALK-5, ALK5, LDS1A, LDS2A, MSSE, SKR4, TGFR-1 | transforming growth factor, beta receptor 1 | MAPK signaling pathway; Cytokine-cytokine receptor interaction; Endocytosis; TGF-beta signaling pathway; Osteoclast differentiation; Adherens junction; Chagas disease (American trypanosomiasis); HTLV-I infection; Pathways in cancer; Colorectal cancer; Pancreatic cancer; Chronic myeloid leukemia. | 249 | 2.00E-66 | 426 |
| TRAF2 | hsa:7186 | IPI00030278 | TRAF2, MGC:45012, TRAP, TRAP3 | TNF receptor-associated factor 2 | MAPK signaling pathway; Protein processing in endoplasmic reticulum; Apoptosis; Osteoclast differentiation; RIG-I-like receptor signaling pathway; Adipocytokine signaling pathway; Hepatitis C; Herpes simplex infection; Pathways in cancer; Small cell lung cancer. | 126 | 2.00E-29 | 501 |
| TRAF6 | hsa:7189 | IPI00743663 | TRAF6, MGC:3310, RNF85 | TRAF6, MGC:3310, RNF85 | MAPK signaling pathway; Ubiquitin mediated proteolysis; Endocytosis; Osteoclast differentiation; Toll-like receptor signaling pathway; NOD-like receptor signaling pathway; RIG-I-like receptor signaling pathway; Neurotrophin signaling pathway; Pertussis; Leishmaniasis; Chagas disease (American trypanosomiasis); Toxoplasmosis; Tuberculosis; Hepatitis C; Measles; Herpes simplex infection; Pathways in cancer; Small cell lung cancer. | 124 | 1.00E-28 | 522 |

*^a^*:Further information of these proteins can be found in File S10.

*^b^*: The information of these eight proteins are summarized from Kegg: www.genome.jp/kegg/
